# Airway sizes and proportions in children quantified by a video-bronchoscopic technique

**DOI:** 10.1186/1471-2466-6-5

**Published:** 2006-03-08

**Authors:** Ian B Masters, Robert S Ware, Paul V Zimmerman, Brian Lovell, Richard Wootton, Paul V Francis, Anne B Chang

**Affiliations:** 1 School of Medicine, Discipline of Paediatric and Child Health, University of Queensland, Herston 4029, Brisbane, Australia; 2 Department of Respiratory Medicine, Royal Children's Hospital, Herston 4029, Brisbane, Australia; 3 School of Population Health, The University of Queensland, Herston 4006, Brisbane, Australia; 4 Department of Thoracic Medicine, The Prince Charles Hospital, Rode Rd, Chermside 4032, Brisbane, Australia; 5 University of Queensland, School of Information Technology and Electrical Engineering, St Lucia 4072, Brisbane, Australia; 6 University of Queensland Centre for Online Health, Level 3 Foundation Building, Royal Children's Hospital, Herston 4029, Brisbane, Australia

## Abstract

**Background:**

A quantitative understanding of airway sizes and proportions and a reference point for comparisons are important to a pediatric bronchoscopist. The aims of this study were to measure large airway areas, and define proportions and factors that influence airway size in children.

**Methods:**

A validated videobronchoscope technique was used to measure in-vivo airway cross-sectional areas (cricoid, right (RMS) and left (LMS) main stem and major lobar bronchi) of 125 children. Airway proportions were calculated as ratios of airways to cricoid areas and to endotracheal tube (ETT) areas. Mann Whitney *U*, T-tests, and one-way ANOVA were used for comparisons and standard univariate and backwards, stepwise multivariate regression analyses were used to define factors that influence airway size.

**Results:**

Airways size increased progressively with increasing age but proportions remained constant. The LMS was 21% smaller than the RMS. Gender differences in airways' size were not significant in any age group or airway site. Cricoid area related best to body length (BL): cricoid area (mm^2^) = 26.782 + 0.254* BL (cm) while the RMS and LMS area related best to weight: RMS area (mm^2^) = 23.938 + 0.394*Wt (kg) and LMS area (mm^2^) = 20.055 + 0.263*Wt (kg) respectively. Airways to cricoid ratios were larger than airway to ETT ratios (p = 0.0001).

**Conclusion:**

The large airways progressively increase in cross sectional area size, maintain constant proportional relationships to the cricoid and are gender independent across childhood. Anthropometric factors (body length and weight) are significantly related to but only have weakly predictive influences on major airway size. The cricoid is the most suitable comparator for other airway site measurements. These data provide for quantitative comparisons of airway lesions.

## Background

In pediatric respiratory medicine airway malacia disorders and disorders of size and shape are common [1] yet there are no in-vivo quantitative measurement studies of these disorders. The semi-quantitative photogrammetric techniques that have been published regarding these disorders [2,3] are limited by a number of factors [4], and at best, have tended to underestimate the change being measured. Some of these limitations have now been overcome by our new quantitative technique for measurement of airway lumen: the colour histogram mode technique [5]. However when malacia is identified it can only have relevance if there is a reference point. Similarly as bronchoscopic techniques evolve roles in research of areas such as wheezy infants and even endobronchial challenge testing [6], the ability and capacity to measure luminal changes and an understanding of airway proportions and an appropriate comparator site for measurements becomes essential.

The cricoid with its easily accessible position, relative resistance to distortion with pressure change, relatively constant shape at its outlet [7] and its ease of identification all contribute to make it the ideal site for distal airway comparisons. Indeed its size being similar to that of age appropriate endotracheal tube (ETT) potentially provides an additional way for comparisons [5]. However there are no in-vivo data: the available data on these subjects being limited to autopsy work [8-12]. When lung function tests have been used to assess airway size and associated governing factors, they only offer unquantified nominal statements or inferences on airway cross-sectional area within compartments of the airways as exemplified by the gender differences in lung function which have been almost universally reported as having resulted from small airways [13-17]. In addition to making bronchoscopic assessments of size, the ability to understand factors contributing to specific airway site sizes from anthropometric factors would obviously be very useful to the clinician and researcher alike.

As there is little data dealing with the issues of actual quantified airway sizes in terms of cross-sectional area measurements and airway proportions, the aims of this study were to: (i) define the sizes and the relationship of the large airways to their respective cricoids and (ii) define factors that influence these relationships. We hypothesized that that the large airways undergo proportionate growth when compared to the cricoid as a reference point and that gender and the usual childhood anthropometric factors (age, height or body length, weight, gender) influence airway size.

## Methods

### Patients

Children aged 10 years or younger referred for bronchoscopic assessments of chronic cough symptomatology were invited to participate in this study. Anthropometric factors body length (BL) was measured to nearest 0.1 cm from crown to heel (Kiddimetre, Raven Equipment, Essex) and head circumference (HC) (nearest 0.1 cm) and weight (Wt) measured electronically (nearest 0.01 kg) immediately before the bronchoscopy. Exclusion criteria were structural airway abnormalities or significant tracheomalacia. An a-priori definition of significant tracheomalacia was defined as any shape abnormality in the trachea that was = 40% of the cricoid area. This "cut off " was based on the "normal data" derived from autopsy work of Butz [10] where by mid to lower third tracheal flattening was commonly found in infants. Parental consent was obtained and the Research and Ethics committee of the hospital approved the project.

### Bronchoscopic procedure and equipment

The bronchoscopy was carried out using our combined spontaneously breathing gaseous general and local anaesthetic technique [18,19]. The bronchoscope entered the airway through the nose via a right-angled swivel port in the facemask. Images were recorded sequentially from the: (i) right main stem bronchus (RMS), (ii) left main stem bronchus (LMS), (iii) right bronchus intermedius (RBI), (iv) right lower lobe bronchus (RLL), (v) left upper lobe bronchus (LUL), (vi) left lower lobe bronchus (LLL), and (vii) cricoid. The image acquisition time was less than 5 minutes and it was followed by our routine bronchoscopic procedure.

### Methodology of measurement of airway size: Colour Histogram Mode Technique (CHMT) or its visual modification [5]

Using digitally recorded images, the CHMT [5] and the magnification characteristics of an Olympus 3.9 videobronchoscope [4] the cross-sectional area of the airways of interest were measured at end expiration with the tip of the bronchoscope withdrawn and held 10 mm from the "touched" defined carina or assessment site [5]. The RBI assessment site was the RUL inferior margin; the RLL, the right middle lobe (RML) inferior margin and LUL and LLL at their sub-division carina. The RML and the right upper lobe (RUL) could not be measured at this 10 mm distance and were excluded. Images could not be obtained at all sites in some children. The largest of three areas within 10% of each other was accepted as the measurement for that site. Airway proportions were derived as a ratio (airway site area: cricoid area). The airways areas were also compared to the area of an age appropriate endotracheal tube calculated from the external diameter of the tube. Cole's formula (Age/4 + 4) was used to calculate the age appropriate endotracheal tube (ETT) [20]. Age fractions were grouped and coded across 0.5 mm ranges eg. an age range of 3.25 to 3.749 was coded as a 3.5 tube.

### Statistics

Children were grouped into 3 age groups (Group I = 2.5 years, Group II >2.5 to = 5 years and Group III > 5 to = 10 years) for comparative purposes. Mann-Whitney *U *test was used for age comparisons. The airway area data was normally distributed (Kolmogorov-Smirnov); therefore unpaired T tests were used for group comparisons. One-way ANOVA with Tukey correction was used for the age group and sidedness assessments. Univariate standard linear and multivariate backward, step-wise regression analyses were used to develop models that predict determinants of airway size.

## Results

One hundred and twenty five children were enrolled of whom 87 were males and 38 females. The median (range) age of the overall group was 2.05 (0.13–9.80) years and there were no significant differences between the median ages of males 1.91 (0.13–9.25) years and females 2.51 (0.18–9.80) years (p = 0.30) or within any age grouping with p values = 0.59, 0.66 and 0.19 for the 3 age groups I, II and III respectively. All children in this cohort had chronic cough and none had significant airway lesions.

### Airway Size: Cross-sectional area

The age group related mean ± SD cross-sectional area measurements of the cricoid; main stem and lobar bronchi of the bronchial tree are shown in Table 1.

The numbers were unequal at each site because recording measurements 10 mm from the object were not always logistically possible. The LMS area measurements were significantly smaller than the RMS in all age groups. The all groups mean ± SD LMS area of 23.77 ± 8.66 mm^2 ^was significantly smaller than the RMS of 29.95 ± 8.99 mm^2 ^(p = 0.001). This relationship difference was maintained in all age groups: Group I: RMS = 27.790 mm^2^, LMS = 23.092 mm^2^, p = 0.001; Group II: RMS = 30.657 mm^2^, LMS = 22.571 mm^2^, p = 0.004 and Group III: RMS = 36.512, LMS = 27.390 mm^2^, p = 0.005. The mean whole group (ie. all groups combined) LMS: RMS area ratio was 0.79; that is the LMS was on average 21% smaller than the RMS. These relationships were 0.83, 0.74 and 0.75 for the youngest to oldest age groups respectively. There was no gender difference in airway sizes generally or at any of the specific sites or for any particular age group as exemplified by the total group mean ± SD cricoid size for males being 36.035 ± 8.98 mm^2 ^and 35.512 ± 6.19 mm^2 ^for females (p = 0.7).

### Potential factors influencing airway size: Regression analyses

Univariate analyses relating age, body length, weight, body mass index, head circumference (HC) and gender with airway cross-sectional areas of the cricoid, RMS and LMS are presented in Table 2. Body length had the highest correlation with cricoid, RMS and LMS with p-values of 0.006, 0.0001 and 0.032 respectively (Table 2).

**Figure 1:**
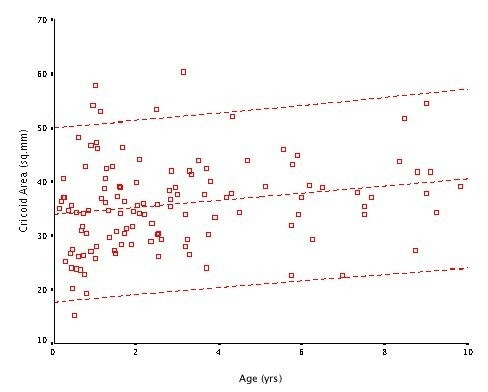
Cricoid area (sq. mm) plotted against age (yrs) with the line of best fit and 95% CI lines derived from univariate analyses.

**Figure 2:**
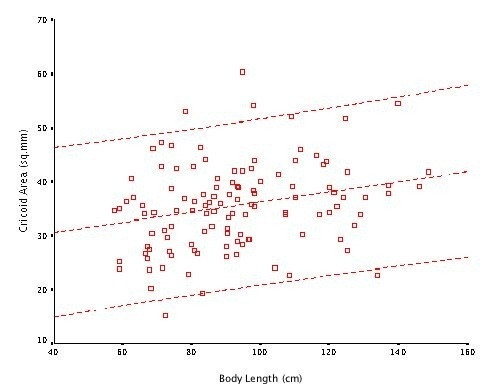
Cricoid area (sq. mm) plotted against body length (BL) with the line of best fit and 95% CI lines derived from univariate analyses.

**Figure 3:**
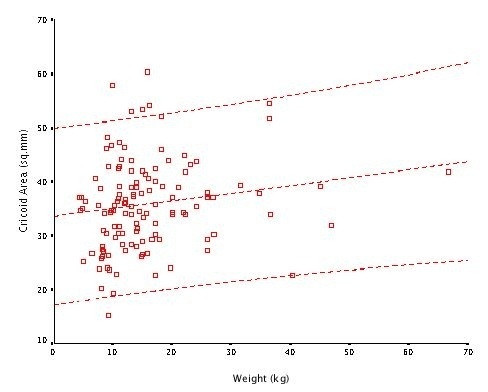
Cricoid area (sq. mm) plotted against weight (Wt) with the line of best fit and 95% CI lines derived from univariate analyses.

The univariate relationships and scatter of the data is exemplified in the regression graphs with the line of "best fit" ± 95%CI for cricoid area plotted against age, body length and weight in Figures 1,2,3. Despite the significant relationships with body length for all airway sites revealed in the univariate analysis, the multivariate backward step-wise regression revealed body length to be the important determinant of cricoid area while weight was the best determinant of RMS and LMS areas (Table 3). The univariate variables that did not have a significant predictive relationship with the outcome variable were removed from the multivariate analysis. The multivariate regression equation for the cricoid area was: cricoid area (mm^2^) = 26.782 + 0.254*BL (cm). The regression equation for the RMS area was: RMS area (mm^2^) = 23.938+ 0.394*Wt (kg) while the equation for the LMS area was: LMS area (mm^2^) = 20.055+ 0.263* *Wt (kg).

### Airway proportions

The mean ± SD airways area to cricoid area ratios (ACR's) for each site in the airway and each age group are shown Table 1. There was no significant difference between the airway proportions across the childhood age ranges (p = 0.48). In addition the proportions of the airways within their respective lungs (right p = 0.16 and left p = 0.49) remained constant across the childhood period

### Airway to Cricoid area Ratio (ACR) and Airway to Endotracheal tube area Ratio (AER)

The mean ± SD relative proportions of the bronchial divisions area to their respective cricoid area and to the area of the age appropriate endotracheal tube area are shown in Table 4. The ACRs were larger than the AERs at all sites and their respective differences were statistically significant at all sites.

## Discussion

This is the first study reporting detailed in-vivo airway cross-sectional area measurements from cricoid to lobar bronchial divisions and area based airway proportion measurements from children using a validated bronchoscopic methodology. We found that body length and weight but not gender influenced the size of major airways measured. In addition we found that there were no gender differences in large airway sizes as assessed by cross sectional area measurements and the cricoid to be most suitable for comparative measurements as ETT derived areas underestimated comparative assessments.

In children there are no in-vivo studies that have determined major airway sizes and proportions to the level of the lobar bronchi. Existing data on this subject is limited to autopsy studies involving small numbers of subjects and not surprisingly there is a wide scatter of values [9-12]. Despite the considerably larger numbers of subjects in our study we also found a wide scatter in sizes of the large airways but have affirmed that the LMS is significantly smaller that the RMS (79% of RMS area) and that this relationship remains constant across childhood. This latter issue has not been previously described even though there have been suggestions of such from autopsy and computerized tomography (CT) based studies [10,21,22]. With respect to the anthropometric factors that influence airway size, the multivariate analyses (Table 3) revealed significant but only very weak predictive associations with the low adjusted r^2 ^and β values. This indicates that either additional factors or factors other than those examined are likely to be important. The children in this study were relatively healthy without histories of nutritional state disorders, prematurity or growth-retarded birth, severe neonatal and acquired lung disease that are known to interfere with somatic lung growth. We did not analyse for other potential confounders such as drug and or cigarette smoke exposure in pregnancy. However it is more likely that these effects would influence airway size when there is somatic growth effects such as growth retardation, whereas in contradistinction lung function assessments might be affected by exposure without somatic growth effects [23,24].

Airway proportion measurements from cadaver studies suggest that the airways progressively and proportionately reduce size from the central to peripheral airways in both children and adults [8-10,25]. However the numbers in these studies were small. Our study with far greater numbers of subjects confirms these findings and indeed shows the large airway proportions remain constant across the whole infancy and childhood periods. Combining these factors with the lack of gender differences in size of the large airways and therefore proportions, lends support to the small airways being the likely sites of gender related differences in lung function in children [13-17]. This raises the possibly of gender differences in factors governing growth of the small airways. However these interpretations require some caution, as even though we accounted for the dynamic respiratory cycle changes by measuring the cross sectional area at end expiration and the proportions remained constant across age groups, we did not control for lung volume, and we did not make independent confirmatory assessments of growth of the large airways using volumetric and length measurements. In addition to this new information, these data provide the clinician and researcher with both absolute values and greater perspective of the range of changes that could be expected during transitions from the trachea to mainstem and to lobar bronchi respectively.

In relating airway proportions a suitable denominator, as the comparator is necessary. We used the cricoid as the comparator as it is easily accessible and identifiable, has a relative resistance to distortion with pressure change, and has a relatively constant shape at its outlet [7]. The importance of a comparator is also evident when one describes measurements of tracheomalacia, bronchomalacia and or airway stenoses. The significant differences between the ACR and AER indicate or support the use of the cricoid area as a reasonable comparative point for airway lesions in that it is less likely to underestimate proportions than the ETT as a comparator. This level of underestimation is however expected as Cole's formula was designed for safety of placement of the ETT through the larynx. It is therefore likely that these differences are of no clinical consequence and in fact, would equate to approximately one half of an age appropriate ETT size. Consequently, the ETT size assessment could provide a useful and acceptable adjunct to measurements as has been described for ETT use in upper-airway lesions in the past and particularly when the cricoid is itself involved in pathology [26].

A potential limitation of this study pertains to patient selection and using symptomatic patients as "normals". While it is unlikely that it will ever be ethically acceptable for normal children to undergo an invasive bronchoscopic procedure to resource this type of data for comparative purposes, it is also equally unlikely that airways' sizes measured in these children are appreciably different to that of normal children. Indeed, to date there are no data dealing with the large airways to suggest otherwise. In addition, the children in our cohort were otherwise well (those with intercurrent illness were excluded); the cricoid areas were larger than the area predicted from ETT size and our data is similar to the values obtained from cadaver samples.

Another potential limitation relates to the methodology of measurement, in particular our using a 10 mm viewing distance to measure angulated sites such as the RML and RUL and our ability to precisely define this 10 mm distance. Indeed we excluded these sites from our measurements because using even shorter viewing distances to overcome angulation difficulties results in even greater magnification, thus rendering the image outside the limits of measurement. The use automated distortion correction along with repeated and sequential assessments while approaching and withdrawing the bronchoscope from the object of interest using the applications of optic flow techniques [27] and its recent advances [28,29] that could be applied to bronchoscopic assessments, might overcome these issues and measurement failures and offer an even more detailed assessment of airway anatomy. This would potentially include a three-dimensional (3-D) reconstruction image of the accessible airways. Despite these current limitations, the advantages of this technique exceed those of reconstruction CT and or CT "virtual bronchoscopy" where their capacity to localize specific sites such as the distal end of the cricoid and the origins of lobar bronchi are limited. In addition when used in this context, the radiation doses for extensive, localised and particularly for repeated assessments remain a considerable safety concern to both clinical and research work in children [30,31].

## Conclusion

We conclude that large airways of children grow progressively with increasing age but their proportions with respect to the cricoid remain constant until at least 10 years of age. Gender does not influence cricoid or RMS or LMS size in childhood. The regression equations indicate that anthropometric factors of body length and weight are significantly related to but only have weakly predictive influences major airway size. The cricoid area is a suitable comparator or reference point for comparisons of lower airway sizes and lesions.

## List of abbreviations

RMS = right main stem bronchus

LMS = left main stem bronchus

RBI = right bronchus intermedius

RLL = right lower lobe bronchus

LUL = left upper lobe

LLL = left lower lobe

ACR = airway to cricoid area ratio

AER = airway to endotracheal tube area ratio

ETT = endotracheal tube

CHMT = colour histogram mode technique

CT = computerized tomography

## Competing interests

The author(s) declare that they have no competing interests.

## Authors' contributions

IBM carried out the vast bulk of the concept of design, data acquisition, data analysis, planning and writing of the article.

RSW carried out the supervision of the statistics and its analysis support.

PVZ participated in concept design and planning and writing of the article.

BL participated in the development of concepts of measurement and planning and writing of the article.

RW participated in the development of the concepts of measurements and writing of the article.

PWF participated in the planning and writing of the article.

ABC provided overall supervision of the project design, planning, data analysis and writing of the article.

## Pre-publication history

The pre-publication history for this paper can be accessed here:



## Figures and Tables

**Table 1:** Airway sizes and airway to cricoid ratios (ACR) at specific sites for overall and specific age groups across childhood: mean ± SD cross sectional area (CSA, mm^2^) and mean ± SD airway to cricoid ratios (ACR).

	**Cricoid**	**RMS**	**RBI**	**RLL**	**LMS**	**LLL**	**LUL**
**Overall Group**							
**n**	125	114	100	90	108	97	89
**CSA mm**^2^	38.5 ± 8.26	29.95 ± 8.99	25.86 ± 8.70	18.54 ± 6.86	23.77 ± 8.66	16.53 ± 6.60	14.86 ± 6.35
**ACR**		0.85 ± 0.26	0.73 ± 0.24	0.55 ± 0.23	0.68 ± 0.27	0.48 ± 0.18	0.42 ± 0.18
**Group I (age = 2.5 yrs)**							
**n**	72	67	60	50	64	56	48
**CSA mm**^2^	34.71 ± 8.48	27.79 ± 7.48	23.98 ± 7.52	17.00 ± 5.73	23.09 ± 7.95	15 ± 6.62	12.75 ± 4.60
**ACR**		0.83 ± 0.23	0.71 ± 0.24	0.53 ± 0.22	0.70 ± 0.27	0.47 ± 0.20	0.37 ± 0.13
**Group II (age >2.5 = 5 yrs)**							
**n**	28	28	22	21	24	22	21
**CSA mm**^2^	36.89 ± 7.94	30.66 ± 10.07	26.80 ± 10.06	18.78 ± 6.58	22.57 ± 9.00	17.58 ± 6.53	15.64 ± 6.40
**ACR**		0.84 ± 0.23	0.72 ± 0.25	0.54 ± 0.20	0.64 ± 0.29	0.49 ± 0.19	0.42 ± 0.18
**Group III (age >5 = 10 yrs)**							
**n**	25	19	18	19	20	19	20
**CSA mm**^2^	37.81 ± 7.68	36.51 ± 9.31	30.98 ± 8.83	22.35 ± 8.53	27.40 ± 9.90	19.80 ± 5.54	19.10 ± 7.77
**ACR**		0.92 ± 0.29	0.80 ± 0.25	0.63 ± 0.26	0.66 ± 0.22	0.51 ± 0.13	0.53 ± 0.21

**Table 2:** Univariate associations between potential predictors (Age, Wt, BL, HC, BMI, Gender) and outcomes (cricoid, RMS and LMS cross-sectional areas).

**Outcome**	**Predictor**	β **value**	**Unadj r**^2^	**F statistic**	**p-value**
**Cricoid area (mm**^2^**)**	Age (yrs)	0.205	0.042	5.432	0.021
	Wt (kg)	0.163	0,026	3.375	0.069
	BL (cm)	0.254	0.065	8.00	0.006
	HC (cm)	0.192	0.037	4.297	0.040
	BMI (kg/m^2^)	-0.004	0.000	0.002	0.962
	Gender	-0.023	0.001	0.067	0.796
**RMS area (mm**^2^**)**	Age (yrs)	0.378	0.143	18.661	0.0001
	Wt (kg)	0.394	0.155	20.591	0.0001
	BL (cm)	0.383	0.147	18.103	0.0001
	HC (cm)	0.281	0.079	8.944	0.003
	BMI (kg/m^2^)	0.171	0.029	3.163	0.078
	Gender	-0.039	0.002	0.172	0.679
LMS area (mm2)	Age (yrs)	0.245	0.060	6.788	0.010
	Wt (kg)	0.263	0.069	7.849	0.006
	BL (cm)	0.214	0.046	4.745	0.032
	HC(cm)	0,220	0.039	4.931	0.029
	BMI (kg/m^2^)	0.151	0.023	2.308	0.132
	Gender	0.164	0.027	2.943	0.089

**Table 3:** Multivariate associations between potential predictors (Age, Wt, BL, HC,) and outcomes (cricoid, RMS and LMS cross-sectional areas).

**Outcome**	**Predictor**	β **value**	**Adj r**^2^	**F statistic**	**p-value**
**Cricoid area mm**^2^	BL (cm)	0.254	0.056	7.448	0.007
**RMS area mm**^2^	Wt (kg)	0.394	0.147	18.384	0.0001
**LMS area mm**^2^	Wt (kg)	0.263	0.059	6.887	0.010

**Table 4:** Mean airway proportions using cricoid area and age appropriate endotracheal tube size area as the denominators and their comparison p values. ACR = airway/cricoid area ratio and AER = airway/ETT area ratio.

	**Cricoid**	**RMS**	**RBI**	**RLL**	**LMS**	**LLL**	**LUL**
**n**	125	114	100	90	108	97	89
**ACR**	1.00	0.85	0.73	0.55	0.68	0.48	0.42
**AER**	1.20	1.00	0.87	0.60	0.80	0.54	0.47
**p-value**	0.0001	0.0001	0.0001	0.0001	0.0001	0.0001	0.0001
